# Environmental Factors Modulating the Stability and Enzymatic Activity of the *Petrotoga mobilis* Esterase (PmEst)

**DOI:** 10.1371/journal.pone.0158146

**Published:** 2016-06-28

**Authors:** Jose L. S. Lopes, Juliana S. Yoneda, Julia M. Martins, Ricardo DeMarco, David M. Jameson, Aline M. Castro, Nelma R. S. Bossolan, B. A. Wallace, Ana P. U. Araujo

**Affiliations:** 1 Instituto de Física, Universidade de São Paulo, São Paulo, Brazil; 2 Institute of Structural and Molecular Biology, Birkbeck College, University of London, London, United Kingdom; 3 Instituto de Física de São Carlos, Universidade de São Paulo, São Carlos, Brazil; 4 Department of Cell and Molecular Biology, University of Hawai’i at Manoa, Hawaii, United States of America; 5 Biotechnology Division, Research and Development Center, Petrobras, Brazil; CNR, ITALY

## Abstract

Enzymes isolated from thermophilic organisms found in oil reservoirs can find applications in many fields, including the oleochemical, pharmaceutical, bioenergy, and food/dairy industries. In this study, in silico identification and recombinant production of an esterase from the extremophile bacteria *Petrotoga mobilis* (designated PmEst) were performed. Then biochemical, bioinformatics and structural characterizations were undertaken using a combination of synchrotron radiation circular dichroism (SRCD) and fluorescence spectroscopies to correlate PmEst stability and hydrolytic activity on different substrates. The enzyme presented a high Michaelis-Menten constant (K_M_ 0.16 mM) and optimum activity at ~55°C for *p*-nitrophenyl butyrate. The secondary structure of PmEst was preserved at acid pH, but not under alkaline conditions. PmEst was unfolded at high concentrations of urea or guanidine through apparently different mechanisms. The esterase activity of PmEst was preserved in the presence of ethanol or propanol and its melting temperature increased ~8°C in the presence of these organic solvents. PmEst is a mesophilic esterase with substrate preference towards short-to medium-length acyl chains. The SRCD data of PmEst is in agreement with the prediction of an α/β protein, which leads us to assume that it displays a typical fold of esterases from this family. The increased enzyme stability in organic solvents may enable novel applications for its use in synthetic biology. Taken together, our results demonstrate features of the PmEst enzyme that indicate it may be suitable for applications in industrial processes, particularly, when the use of polar organic solvents is required.

## Introduction

The genus *Petrotoga* belongs to phylum Thermotogae, whose members have a distinctive outer membrane expanded at the ends of the cell, usually denominated as “toga”. The bacteria of this genus are Gram-negative rods, anaerobic, non-spore forming, fermentative and thermophilic [1]. To date, seven species of *Petrotoga* have been described and all of them exclusively isolated from oil reservoirs [2–7]. *P*. *mobilis* is able to ferment a wide variety of carbohydrates, including xylan [3], and its optimum growth temperature are between 58–60°C. Such characteristics make this specie a potentially interesting source of thermostable enzymes for industrial and biotechnological applications.

Lipolytic enzymes catalyze the cleavage and formation of ester bonds in the presence of water. Two groups of these enzymes are the lipases (EC 3.1.1.3) and the carboxylesterases (EC 3.1.1.1), which differ in their biochemical properties [8].

Esterases catalyze the hydrolysis and the formation of short chain fatty acid esters, while lipases display maximal activity against water-insoluble long-chain triglycerides [9–12]. They are members of the α/β hydrolase family, which share a characteristic αβ-fold based on eight-stranded mostly parallel β-sheet surrounded on both sides by α-helices. Both types of enzymes have found applications in a variety of fields including oleochemical, pharmaceuticals, agrochemical, bioenergy, and food/dairy industries [13].

Enzymes produced by thermophiles are of considerable interest because of their thermal stability and preserved activity at elevated temperatures. Moreover, they can also possess remarkable stabilities towards common protein denaturants, such as detergents, low and high pH and organic solvents [14]. The use of enzymes in organic solvents offers numerous potential advantages compared to traditional aqueous enzymology, such as higher solubility of hydrophobic substrates, reduced incidence of side reactions found in aqueous media, and reduced microbial contamination [12]. Enzymes active in water miscible solvents are highly desired in biocatalysis where substrate solubility is limited and also when the solvent is desired as an acyl acceptor in transesterification reactions, as in the case of biodiesel production [15].

The structural features and functional properties of enzymes isolated from thermophilic microorganisms have attracted the interest of many research groups. Lipases/esterases with novel properties are often in demand due to the large number of synthesis reactions which these enzymes can catalyze, but for which enzymatic routes are currently not available due to the reaction environment required [16].

In this study, we performed an in silico search for esterase domains in bacterial genomes of species considered exclusive of oil reservoirs, and based on that, we chose a predicted gene coding for a putative esterase in the *Petrotoga mobilis* genome for heterologous expression. The recombinant enzyme was then characterized using a combination of synchrotron radiation circular dichroism, fluorescence spectroscopies, and hydrolytic assays to explore its stability and hydrolytic activity in different environments.

## Materials and Methods

### Materials

All reagents and solvents were analytical grade. Substrates for enzymatic reactions were purchased from Sigma-Aldrich (United Kingdom).

#### Identification of the gene, plasmid construct, protein expression and purification

The identification of PmEst gene in *Petrotoga mobilis* was the result of a large scale screening of bacterial genomes using bioinformatics analyses to identify esterase domains. A search using hmmsearch command from HMMER program [17], using the Hidden Markov Models (HMM) for this domain (PFAM PF00756.15) for searching a database of predicted proteins from 11 genomes of thermophile bacteria. This approach allowed the retrieval of two different proteins in *Petrotoga mobilis* with significant scores.

The synthetic DNA (GenScript, New Jersey, USA) encoding the PmEst gene (according to that deposited on GeneBank (NC_010003.1—locus tag PMOB_RS04940 coding for the protein WP_012208782.1) was inserted into a modified pET28a expression plasmid (Novagen), allowing a production of an N-terminal hexahistidine tagged SUMO fusion protein.

An aliquot (5 mL) of an overnight culture of *E*. *coli* Rosetta (DE3) harbouring the expression plasmid was transferred to 1 L of LB broth containing chloramphenicol (34 μg/mL) and kanamicin (50 μg/mL), and cells were grown at 37°C, under agitation until an OD_600nm_ of 0.6–0.8 was reached. Protein expression was induced by the addition of IPTG (0.2 mM) for 15h, at 20°C. Cells were centrifuged at 6,000g for 40 min at 4°C, suspended in 10 mM sodium phosphate buffer (pH 7.4), and lysed by sonication with 30s- pulses in an 500 Sonic Dismembrator, (Fisher Scientific) for 7 min.

After centrifugation at 15,000g at 4°C for 30 min, the supernatant was loaded onto a 3 mL Ni-NTA superflow column (Qiagen), which was pre-equilibrated and washed with the same phosphate buffer at 4°C. The fusion SUMO-PmEst protein was eluted in the phosphate buffer with 0.5 M imidazole, and then incubated with 200 μg of SUMO-protease. Imidazole and SUMO protein were removed from the reaction by two subsequent chromatographic steps onto a HiPrep 26/10 desalting (GE Healthcare) column, and a Ni-NTA superflow column, respectively. Protein integrity was confirmed on SDS-PAGE 15%.

At the end of the purification, the enzyme was lyophilized and suspended in 10 mM sodium phosphate buffer (pH 7.4) prior to use. Protein concentration was measured by the absorption at 280 nm, using the extinction coefficient of 37,025 M^-1^cm^-1^.

#### Enzyme activity assays (pH, temperature)

The activity of PmEst was monitored continuously at 405 nm in a Cary 60 spectrophotometer (Agilent Technologies) by measuring the release of *p*-nitrophenol molecules hydrolyzed from the hydrocarbon chains at different temperatures for 90 s. The reaction was initiated with the addition of the enzyme (500 nM) in 10 mM sodium phosphate buffer (pH 7.4) on 1 mM of each *p*-nitrophenyl esters substrate: *p*-nitrophenylacetate (*pNA*), *p*-nitrophenylbutyrate (*p*NB), *p*-nitrophenyloctanoate, *p*-nitrophenyldecanoate, *p*-nitrophenyllaurate, *p*-nitrophenylmyristate, and *p*-nitrophenylpalmitate, in acetonitrile (>1%) solution.

To evaluate the optimum temperature of PmEst, this assay was performed in triplicate from 25 to 95°C for 1 min, using 125 μM of *p*NB. To evaluate the thermal stability of the enzyme, PmEst was pre-incubated in a water bath ranging from 25 to 55°C for 30–120 seg, and the activity was immediately measured in triplicate, at 25°C. Moreover, the activity of the enzyme was also monitored in the presence of 10, 20, and 30% of ethanol or propanol at 25°C and 55°C, using the same conditions described above.

#### Bioinformatics analyses

**Fig 1:**
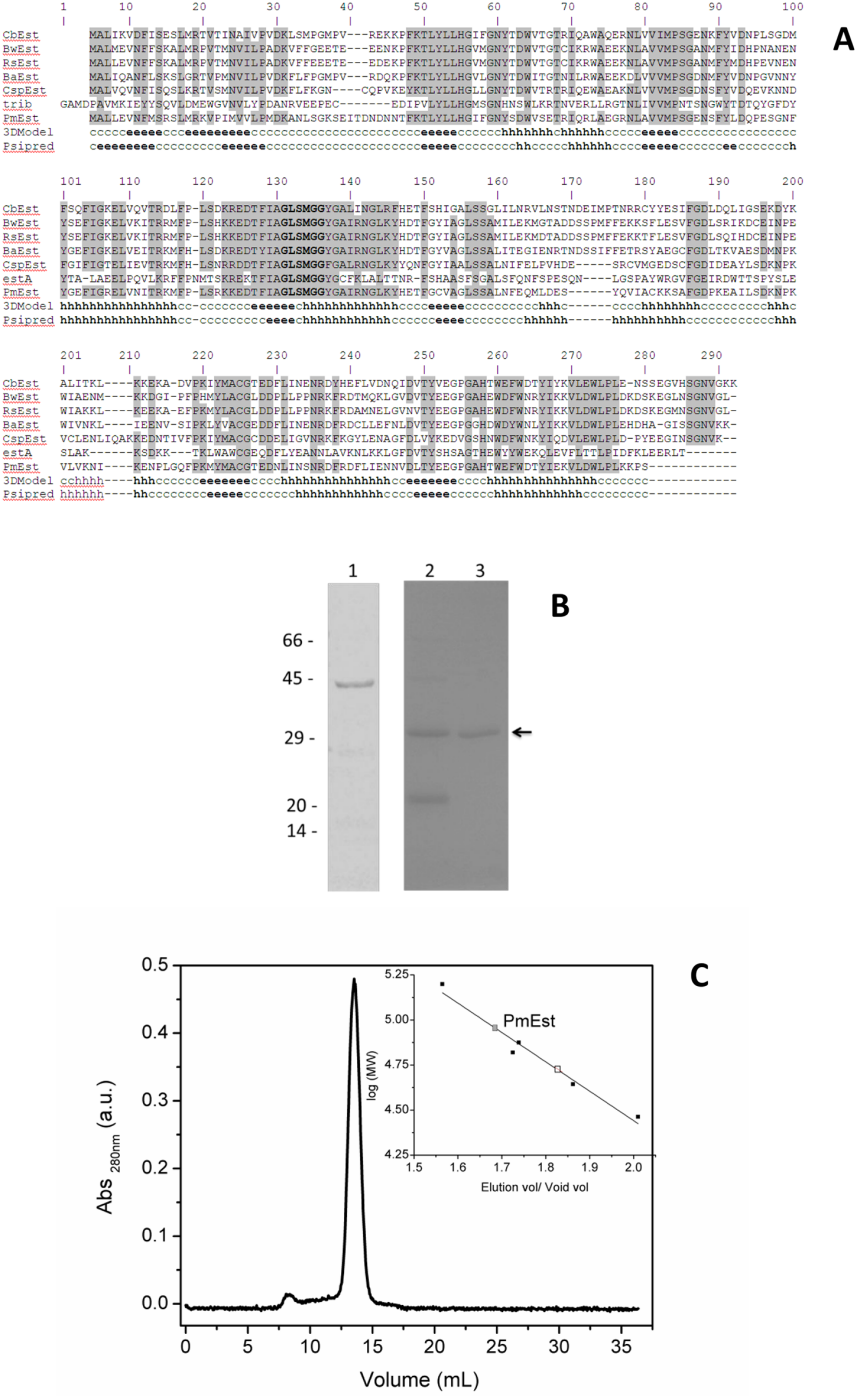
Characterization of the PmEst enzyme. a) Sequence alignment between PmEst and esterases from other organisms. Conserved residues among esterases are highlighted in gray, and residues from esterase domains (GxSxGG) are in bold font. Secondary structure prediction of PmEst using Psipred and based on the 3-D homology model. Secondary structure elements are labeled as h, helix; e, extended β-strand; c, disordered/other. CbEst, acetyl esterase from *Clostridiaceae bacterium* GM1; BwEst acetyl esterase from *Blautia wexlerae*; RsEst, esterase from *Ruminococcus sp*. From JC304; BaEst, acetyl esterase from *Bacillus akibai*; CspEst, esterase from *Coprobacillus sp*.D7; and estA, esterase (PDB ID: 2UZ0, chain B) from *Streptococcus pneumoniae*. b) SDS-PAGE showing: 1) SUMO-PmEst fusion protein after affinity chromatography on Ni-NTA; 2) fusion protein after digestion with SUMO protease; 3) purified PmEst after rechromatography on Ni-NTA column; on the left, molecular weight markers in kDa; black arrow is indicating PmEst protein; c) Size exclusion chromatography on Superdex 200 10/300 GL column (13 μm average particle size) with MW calibration curve inset: carbonic anhydrase (29 kDa), ovalbumin (44 kDa), bovine serum albumin (66 kDa), conalbumin (75 kDa), and aldolase (158 kDa).

Predictions of secondary structure propensity based on the amino acid sequence of PmEst were performed using the Psipred v3.3 method [18] available on ExPasy portal [19]. A homology model for the three dimensional structure of PmEst was generated using the 4 Phyre2 software [20] and the crystalline structure of estA protein (PDB ID: 2UZ0, chain B) as template; both enzymes share 34% of identity (sequential alignment between the two enzymes is in Fig 1a). Calculation of the secondary structure from the PmEst 3-D structure was performed using the 2Struc server [21].

#### Synchrotron radiation circular dichroism (SRCD) spectroscopy

The SRCD spectra of PmEst (46 μM) in aqueous solution were obtained over the wavelength range of 270–170 nm with 1 nm intervals and 2s dwell time, collecting 3 individual scans at 25°C, using a 0.00507 cm pathlength quartz cuvette (Hellma Scientific). Measurements were made at the CD1 beamline on the ASTRID1 Synchrotron at ISA (Denmark), and at the DISCO beamline on the Soleil synchrotron (France).

Aliquots (0.7 nmol) of PmEst were used to form a dried film of the protein on the surface of a quartz Suprasil plate by the evaporation of the solution under moderated vacuum. The SRCD spectrum of the dehydrated film was collected over the range from 270 to 155 nm, with 1 nm interval and 2s dwell time, at 25°C, taking measurements at four different rotations on the plate (0, 90, 180, and 270 degrees) and 2 scans on each position.

All SRCD data of PmEst were processed using CDTool software [22], which was employed to calculate the average of the individual scans, the subtraction of the corresponding baseline spectra, zeroing, smoothing with a Savitzky-Golay filter, calibration with camphorsulfonic acid, and scaling the final spectra in delta epsilon units, using a mean residue weight of 114. Estimations of the structural content of PmEst based on the SRCD spectra were performed using the Dichroweb server [23] with the SP175 database [24], using the goodness-of-fit test [25] and the Continll [26] algorithm.

#### Tryptophan fluorescence spectroscopy

The steady-state emission spectrum of the intrinsic tryptophan (Trp) residues in PmEst (3 μM) in phosphate buffer (pH 7.4) was measured on an ISS K2 instrument (ISS Inc., Champaign, IL) with excitation performed at 300 nm, using 1 cm pathlength quartz cuvettes (Hellma USA Inc.) and emission spectra recorded from 310 to 450 nm in 1nm intervals, at 25°C, with the temperature controlled via a circulating water bath (Fisher Scientific, Pittsburgh, PA). All slitwidths were 8 nm. Three scans were averaged and the corresponding baseline spectrum was subtracted.

The relative fluorescence quantum yield of PmEst was estimated using N-acetyl-tryptophanamide (NATA) (Sigma-Aldrich) as a standard (*Φ* = 0.14) on an ISS PC1 spectrofluorimeter (ISS Inc., Champaign, IL). The assays were carried out by adjusting the optical density of PmEst and NATA to <0.05 at 300 nm in phosphate buffer (pH 7.4) and using 1 cm pathlength quartz cuvettes, with 300 nm excitation with vertical polarized light, and 8 nm slits. Emission was monitored with total fluorescence intensity (*I*_*‖*_ *+ 2I*_*⊥*_) collected using polarizers at magic angle conditions (parallel excitation and 55° emission).

Acrylamide quenching assays of PmEst tryptophan fluorescence were measured on aqueous solutions with the titration of acrylamide (2.5 μL aliquots, 8 M stock solution) into a 2 mL cuvette containing PmEst (1 μM). Excitation was performed at 295 nm and emission spectra were monitored from 305 to 450 nm in duplicate, through WG320 nm emission filters, at 25°C. The Stern—Volmer quenching constants (*K*_*sv*_) were analyzed using the equation:

$$\frac{F_{0}}{F}\;=\;1+{\text{K}}_{SV}[Q]$$

where *F*_*0*_ and *F* are the fluorescence intensities in the absence and in the presence of acrylamide (quencher, *Q*), respectively.

Excited-state lifetimes of PmEst were measured with frequency domain time-resolved fluorescence on an ISS Chronos fluorometer (ISS Inc., Champaign, IL) and a 300-nm LED as excitation light source, passed through a 295 nm (+/- 10 nm) interference filter (Semrock-Rochester, NY), and p-terphenyl (Sigma-Aldrich) dissolved in spectroscopic grade ethanol (Sigma-Aldrich) as lifetime reference (1.04 ns). The phase shifts and the relative modulations of the emitted light were monitored at 15 light modulation frequencies from 30 to 150 MHz, at 25°C (controlled with a circulating water-bath). The data of the complex decay of the Trp residues in PmEst were analyzed using multiexponential functions (discrete model) [27] and the phasor approach [28–31].

#### Effect of pH, temperature and chemical denaturing reagents

The effect of pH on the secondary structure of PmEst was evaluated by incubating the protein in solutions with either alkaline (10 mM sodium borate, pH 11) or acid (10 mM sodium phosphate, pH 3.0) conditions for 30 min and measuring the conventional CD and fluorescence spectra of each sample.

Thermal denaturation assays were performed by heating the PmEst from 15 to 85°C, in 5°C or 10°C increments, and allowing 5 min equilibration at each temperature. SRCD and fluorescence spectra were collected at each temperature using the same parameters described before, using 3 repeats scans at each temperature. The first and the last individual spectra at each temperature were compared to ensure sample had achieved thermal equilibrium prior to the measurements. After heating to 85°C, samples were cooled back to 25°C, in order to assess the reversibility of the changes caused by the effect of the temperature on the structure of PmEst. For the SRCD experiments, principal component analyses used CDTools software.

The effect of urea and guanidine (GndHCl) on the structure of PmEst was investigated by incubating the protein with each of the chaotropic agents in increasing concentrations (from 0.5 to 7.0 M, in 0.5 M steps) in phosphate buffer (pH 7.4) for 30 min, at 25°C. The aliquots containing 7.0 M of each reagent were dialyzed against phosphate buffer, in order to assess the reversibility of the changes causes by the chemical denaturing reagent.

The thermal stability of PmEst in organic solvents was also evaluated by obtaining the conventional CD spectra of the protein (3 μM) in the same phosphate buffer, which also contained 10% or 20% ethanol or 1-propanol from 20 to 80°C, in 5°C increments, allowing 5 min equilibration at each temperature, using a JASCO J-815 spectropolarimeter (Tokyo, Japan) as an average of 3 scans over the wavelength from 270 to 190 nm, in 1 nm intervals, using a 0.1 cm pathlength quartz cuvette. Baseline subtraction and data processing were also performed using CDTool software.

## Results and Discussion

### Protein expression and purification

The gene PmEst was identified by a search using a HMM of an esterase domain (PFAM PF00756.15) to interrogate a database of predicted proteins from 11 thermophile bacteria. Two proteins from *Petrotoga mobilis* displayed significant scores in this search. The first of them displayed an e-value of 0.006 and was annotated as a carboxylesterase. The second displayed an e-value of 10^−7^ and was annotated as a putative esterase. The second protein produced a better match to an esterase domain and therefore was more likely to display an esterase activity. We named this protein PmEst and decided to produce it in a recombinant system to characterize its activity aimed at potential biotechnological applications.

The sequential alignment of PmEst with esterases from different organism shows a putative conserved domain of an esterase/lipase superfamily between residues 47–150. The most conserved region among these enzymes is located within the residues comprising the esterase domain (Fig 1a). SUMO-PmEst fusion protein (~45 kDa) was expressed in soluble form, allowing its purification by affinity chromatography (Fig 1b). Cleavage of the protein fusion tag using SUMO protease shows formation of a ~30 kDa protein, corresponding to the expected mass of PmEst without the carrier SUMO protein. A second affinity chromatography step allowed the removal of the SUMO. The molecular mass observed by size exclusion chromatography (SEC) from the purified fraction was ~90 kDa (Fig 1c), which suggested that the enzyme PmEst could function as a homo-trimer in aqueous solution. A trimeric state was already observed in the esterases from *Pseudomonas putida* (with a 33 kDa monomer) [32] and esterase 6A from *Mus musculus* (with a 60 kDa subunit) [33], but also a functional tetramer has been reported for estA [34].

### Hydrolytic activity of PmEst

PmEst was able to catalyze the hydrolysis of a variety of esters to alcohols. In this case, the acyl *p*-nitrophenyl esters with acyl chains ranging from 2 to 16 carbons were used, and the product (*p*-nitrophenol) was monitored at 405 nm as an indirect measurement of the PmEst activity. The assays were conducted with substrates of different acyl chain sizes, but PmEst was more active on *p*NB, presenting a high Michaelis constant (K_M_ = 0.16 mM). Values reported for esterase 6A and E2 esterase [35] with 4-nitrophenyl hexanoate and beta-naphthyl butyrate, were 44.4 mM and 0.36 mM, respectively. The kinetic parameters of PmEst for *p*NA and *p*NB substrates are listed on Table 1.

**Table 1:** Kinetic parameters of the hydrolytic activity of PmEst.

Substrate	V_max_	*K*_M_(mM)	*k*_cat_ (s^-1^)	*k*_cat_/*K*_M_ (s^-1^. mM^-1^)
*p*NA	2.92±0.12	1.43±0.17	29.2±1.2	20.5±1.6
*p*NB	1.40±0.03	0.16±0.01	14.1±0.29	88.1±6.0

**Fig 2:**
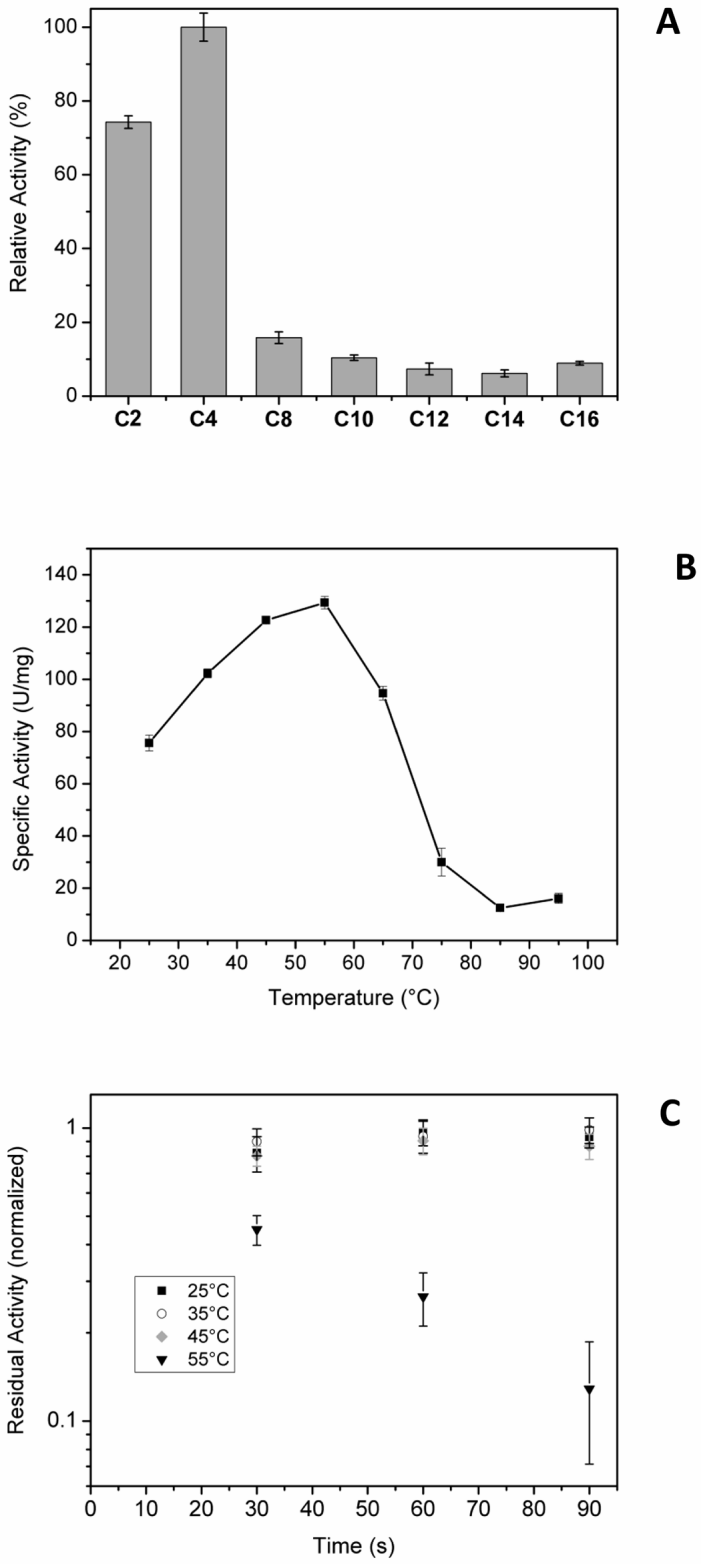
Esterase activity assays. a) The relative hydrolytic activities of PmEst on substrates with different acyl chain sizes were calculated with respect to C4. b) Determination of the optimum temperature of PmEst on *p*NB, and c) Residual activity normalized as a function of pre-incubation time at 25, 35, 45 and 55°C. PmEst was inactive when pre-heated at temperatures higher than 55°C. Error bars represent the standard deviations from the triplicate measurements.

However, the relative activity of PmEst on long-acyl chains substrates was negligible (Fig 2a). A common behavior of esterases is a substrate preference towards short-to medium-length acyl chains (C2–C8) rather than long acyl chains. For example the esterase SAestA, purified from *Salinispora arenicola* [36], and esterase 6A show preferences for C6 esters [33]. In our particular case, there is a strong preference for very short-length chains (C2-C4).

The optimum temperature of PmEst was determined to be ~55°C (Fig 2b), when measured with incubation of the enzyme for 30 s at each condition. Mesophilic enzymes are optimally active between 25 and 50°C, while the thermophilic enzymes are usually optimally active between 60–80°C [37]. This observation would classify PmEst in the interface of a thermo-mesophilic enzyme. However, experiments were also performed with pre-heated PmEst samples (with different incubation times) to gain more insights into the thermal stability of the enzyme.

Fig 2c shows the residual esterase activity as a function of pre-incubation time at 25, 35, 45 and 55°C. For 25, 35 and 45°C the activity remains practically constant during 90 seconds of incubation, however at 55°C, the activity was reduced by half in 30 seconds.

Therefore, although PmEst is in principle very active at elevated temperatures, with an optimum activity at temperature ~55°C, its activity in aqueous solution at >45°C is not long-lasting. Thus, these observations suggest that PmEst is not very stable at high temperatures, being most likely a mesophilic enzyme. One of the reasons for this may be that the bacteria *Petrotoga mobilis* can be found in shallow reservoirs, in which the optimum growing temperature is not very high (~50°C). Indeed, Lien et al. [3] described the *Petrotoga mobilis* sp. nov. from a North sea oil-production well, where they found that the optimum growth occurs between 40 and 60°C.

### Structural information on PmEst

The amino acid sequence of PmEst exhibited up to 60% identity with esterases from other organisms, as shown in Fig 1a. Homology modelling of PmEst suggest this hydrolytic enzyme may be organized in a combination of short α-helices (33% helix content) interspersed for several elements in β-strand (17% content), with a majority of residues in disordered or turns structure (50%). The homology model of PmEst is in agreement with the prediction of an α/β protein, assuming a folding very similar to that observed for estA and other esterases (Fig A in S1 File).

**Fig 3:**
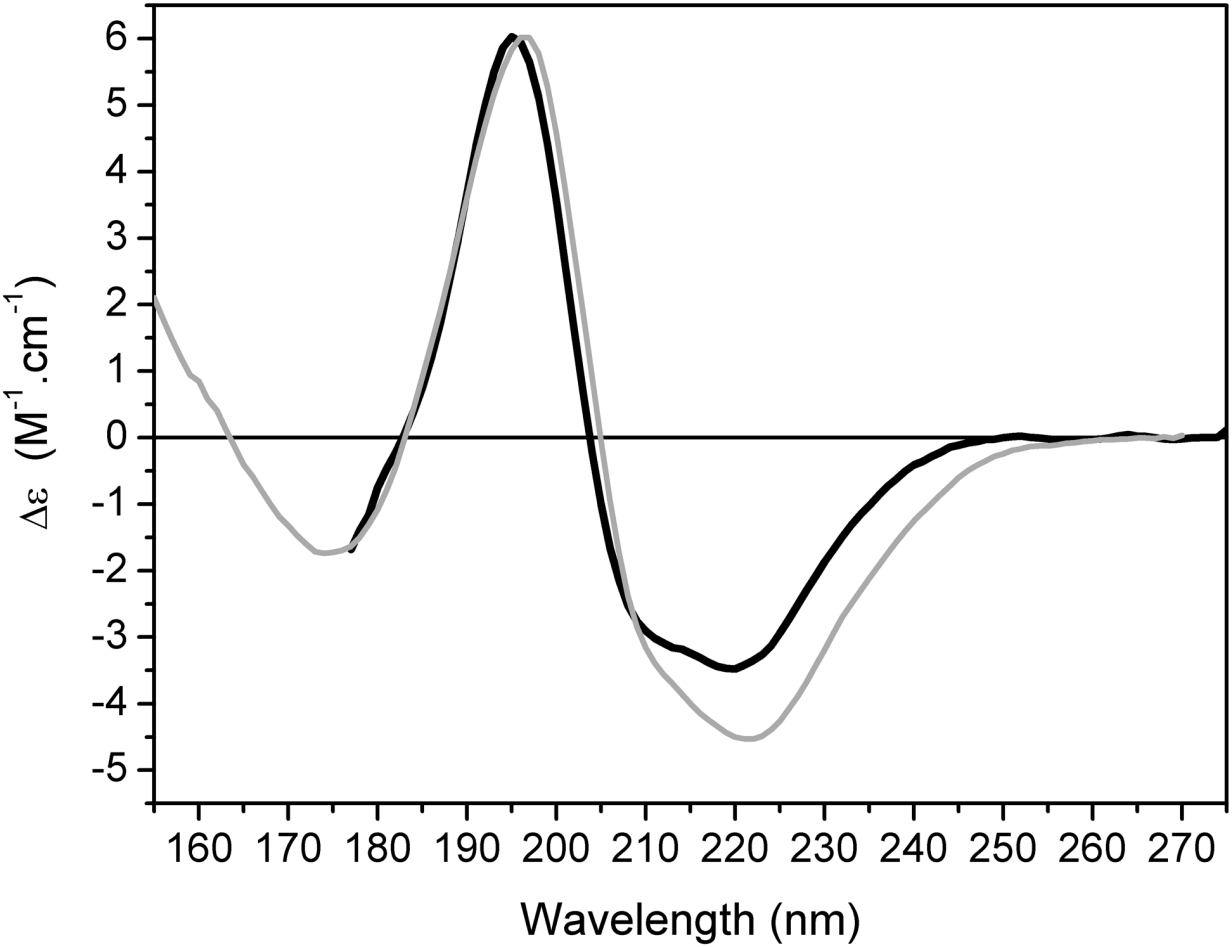
Structural properties of PmEst. SRCD spectra of the enzyme in aqueous solution (black) and in dehydrated films (gray).

Synchrotron radiation circular dichroism analyses on PmEst in aqueous solution confirm the protein contains both helical and strands elements (Fig 3), due to the presence of the two minima at the region of 222 and 208 nm, and the positive maximum at 196 nm that are characteristic spectral bands for α-helices [38–39], and the additional minimum at the vacuum-UV region at 175 nm, which is a band seen in proteins containing β-sheets [40–41]. Moreover, the structural content of PmEst predicted by bioinformatics analyses, and in the 3-D model are in close agreement with the calculated secondary structure of the protein based on its SRCD spectrum in aqueous solution (Table A in S1 Table). The enzyme PmEst also retains this structural content preserved when in a partially-hydrated film deposited on the surface of a quartz plate, preserving the spectral band positions as in aqueous solution, whilst showing more clearly the minimum at ~175 nm (which is more clearly visible when the (non-chiral) absorption due to water molecules in solution has been removed).

Additionally, the fluorescence emission maximum of PmEst in aqueous solution at 25°C was centered at 330 nm (S2 File), typical of a folded protein in which the aromatic residues are buried inside a hydrophobic core, avoiding contact with water molecules. In this condition, the relative fluorescence quantum yield of PmEst was estimated at 0.11 and its acrylamide quenching constant (*K*_*SV*_) was 4.1 M^-1^ (while this value for free NATA was estimated at 20.1 M^-1^), in agreement with the buried Trp residues in the PmEst 3-D structure (Trp residues are labeled in PmEst homology model, S1 File).

### The stability of PmEst to external factors

The native SRCD spectrum of PmEst was not altered when the enzyme was incubated at acid conditions. However, a significant spectral change was observed when the enzyme was incubated at alkaline solutions (pH 11), as shown in S3 File, corresponding to an increase in disordered structure, and thus indicative of denaturation. The deprotonation of acidic and basic groups on proteins at alkaline pH might be affected some of the ionic bonds between charged side chains of amino acids that contribute for the native fold of the enzyme. The weakening of this type of interaction might have resulted in the loss of the native fold as observed on the conformational changes on PmEst CD spectrum at elevated pH. Therefore, it appears that alkaline pHs can affect the maintenance of the native folding of the enzyme more efficiently than acid conditions.

**Fig 4:**
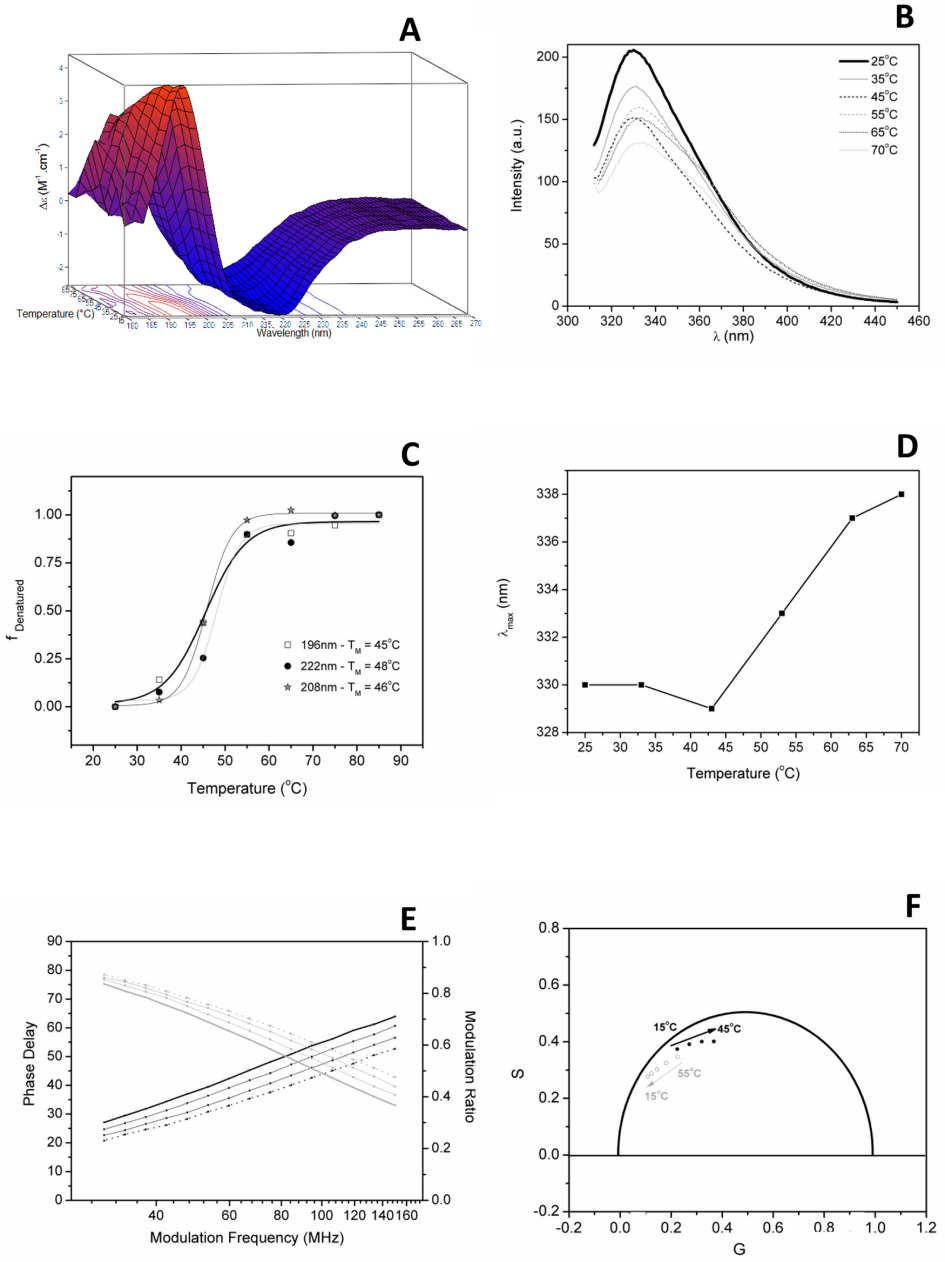
Thermal stability of PmEst evaluated by spectroscopic methods. a) Synchrotron radiation circular dichroism spectra as a function of temperature overlaid in the Z direction; b) emission spectra of PmEst as a function of temperature in sodium phosphate pH 7.4. c) fluorescence emission maxima data as a function of temperature. d) thermal denaturation curves of PmEst, taken from SRCD data at 196, 208, and 222 nm, and e) Phase delay (bottom curves) and modulation ratio (top curves) of the excited state lifetime of PmEst in PBS as a function of temperature, from 15°C (solid) to 45°C (dot) in10°C steps; and f) their respective phasor diagram, showing the point at 55°C is not on the same trajectory of the other point at below temperatures.

In the spectroscopic assays, PmEst once again was shown to be a protein with moderate thermal stability, in agreement with what was observed in the activity assay. A gradual decrease of its ordered content was observed by circular dichroism analysis when PmEst was incubated at increasing temperatures between 15 and 85°C. The native SRCD spectrum of PmEst started to present slight changes ~35°C. Fig 4a shows the 3-D plot for the SRCD spectra of the thermal melting of PmEst at pH 7.0. The melting temperature of PmEst was estimated to be ~47°C (Fig 4b). At high temperatures (~85°C) the SRCD spectrum of PmEst was not typical of a fully unordered/denatured protein. However, there was no hydrolytic activity detected at this temperature.

In agreement with the SRCD data, the redshift in the fluorescence emission maximum of PmEst and a reduction of the fluorescence intensity also occurred as the temperature increased (Fig 4c). Specifically, as the temperature increased, the maximum emission changed to ~340 nm, suggesting a partial exposition of the aromatic residues of PmEst to the aqueous solution. The Tm obtained by SRCD analysis was also confirmed with fluorescence thermal assays (Fig 4d).

At 25°C in aqueous solution, the lifetimes in the excited state of the Trp residues in PmEst, could be fitted with two exponentials with lifetimes values of 5.3 and 1.7 ns, and fractions of 35 and 65%, respectively. Using discrete model analysis, the lifetimes were reduced with an almost linear dependence upon the temperature increase up to 45°C (Fig 4e, values are in Table B in S1 Table), in accordance with the gradual exposure to water molecules observed at the local environment of the Trp residues.

Due to the complexity inherent in analysing the lifetimes of a protein containing multiple (4) tryptophans, the time-resolved fluorescence data were also considered using the qualitative phasor method. The phasor points of PmEst Trp fluorescence fall inside of the universal circle on the phasor diagram (Fig D in S2 File), showing that the excited-state decay of the Trp residues should be described by a multiexponential decay. During the heating process, the points in the phasor diagram presented a trajectory with a clockwise movement, indicating the reduction in the lifetimes of the Trp residues, in agreement with the data in Table B in S1 Table. In Fig 4f, the points in the phasor diagram at different temperatures, revealed a remarkable change in the direction of the trajectory when the proteins was heated from 45 to 55°C, suggesting that the process of protein denaturation starts within this temperature interval. After reaching 55°C, the attempts to cool down the protein in gradual steps back to 15°C does not give the same trajectory of the phasor diagram, showing that the thermal denaturation of PmEst was not a reversible process.

**Fig 5:**
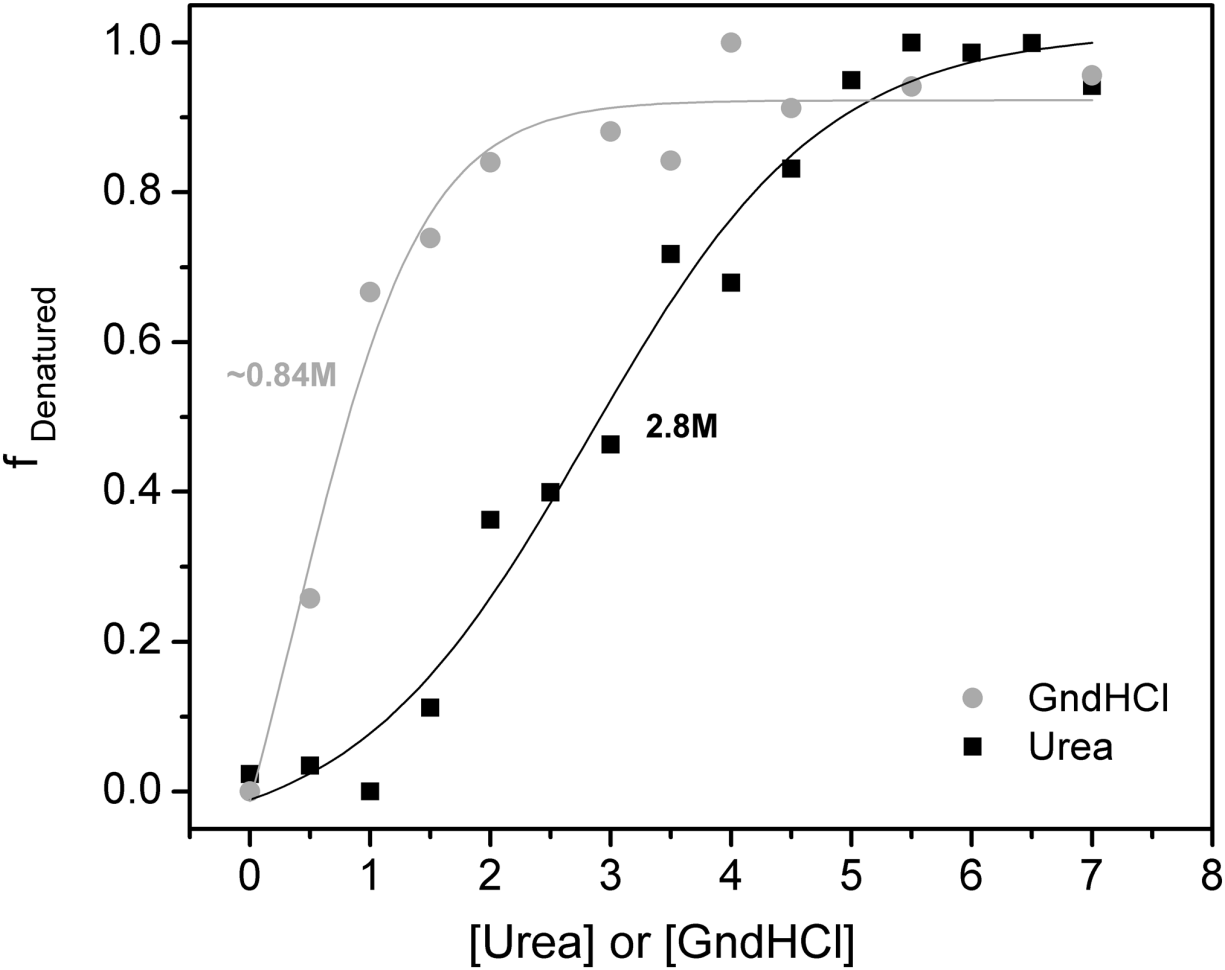
Effect of urea and guanidine on the structure of PmEst. Curves of the chemical denaturation of PmEst with addition of urea (black) and GndHCl (gray), taken from the normalized ellipticity value at 220 nm of the CD spectrum registered at each condition. Sigmoidal fit was performed on each curve, considering two different states for PmEst: native and denatured.

The unfolding of PmEst was also seen at elevated concentrations of urea or guanidine (Fig 5). A complete denaturation of the enzyme occurred in 7 M as final concentration of each chaotropic reagent, confirmed by the red shift of the emission of the Trp residues (to 355 nm), due to the total exposure to the aqueous environment. However, PmEst was more susceptible to guanidine than to urea. The value of chaotropic denaturant corresponding to half-completion of the transition to the denatured state was at 0.8 M for guanidine and 2.8 M for urea. Chaotropic urea usually denatures protein by destabilizing internal non-covalent bonds, weakening intermolecular bonds and overall secondary and tertiary structure, whereas GndHCl is a monovalent salt which may have both an ionic and a chaotropic effect [42], thereby having greater influence on the stability of the enzyme more effectively than does urea.

### Stability and activity of PmEst in polar solvents

**Fig 6:**
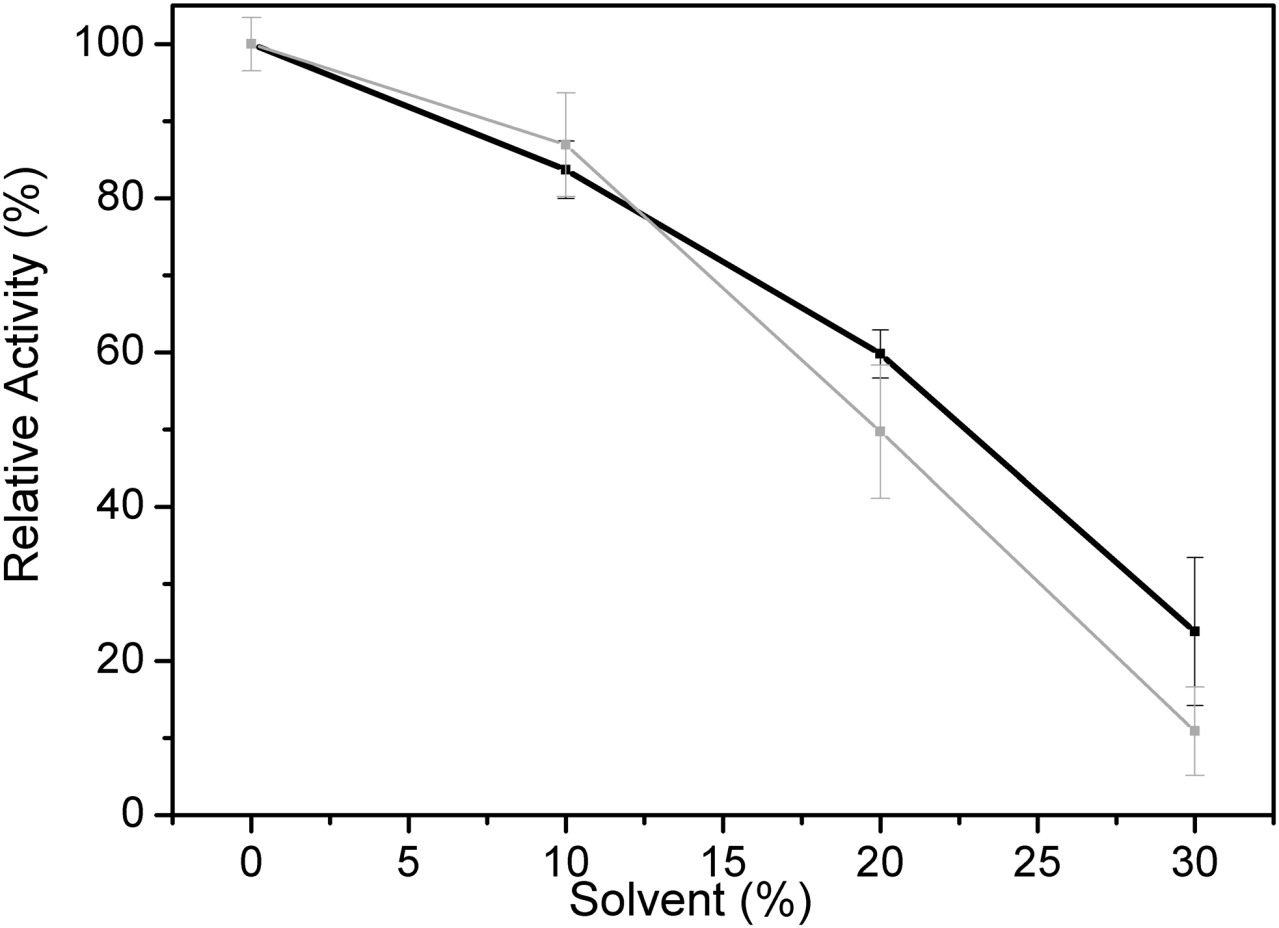
Esterase activity of PmEst in organic solvents. a) Relative activity of PmEst in the presence of 10%, 20%, and 30% of ethanol (black) and propanol (gray). Error bars represent the standard deviation from the triplicate measurements at each temperature.

The hydrolytic activity of PmEst was quite preserved in the presence of modest amounts of organic solvents such as ethanol or propanol. ~85% of the hydrolytic activity (Fig 6) on *p*NB were preserved when 10% of either solvent was added in the aqueous solution. Even in the presence of 20% (final concentration in the solution) of each solvent the enzyme retained some activity, preserving ~55% of its hydrolytic activity. The tolerance of PmEst to water miscible organic solvents is quite significant and could be related to the *Pseudozyma sp*. NII 08165 esterase [15] that preserved 80% of activity in presence of 25% ethanol. On the other hand, other examples of thermostable esterases isolated from *Thermotoga maritima* [43] and *Salimicrobium* [12] showed a dramatic loss of activity (reduction of ~80%) in the presence of 10% ethanol and propanol. This property suggests a potential application of PmEst for industrial processes such as the biodiesel synthesis or in which tolerance to polar organic solvents is required, especially when substrate solubility is limited.

At concentrations of ethanol or propanol in solution above 30%, PmEst still retained about 25% of its activity. In agreement with the activity assays, the SRCD spectra of PmEst showed virtually no changes in the presence of 10 and 20% of organic solvents (Fig A in S4 File). Indeed, PmEst even exhibited a slight enhancement of its thermal stability when the organic solvents were present (Fig B in S4 File). Principal component analyses of PmEst melting showed ~8°C and ~4°C increases in the Tm values for the protein in the presence of 10% and 20% of either organic solvent, respectively.

## Conclusions

In this work, a novel mesophilic enzyme with esterase activity was identified from the genome of *Petrotoga mobilis* and produced in a recombinant form. PmEst appears to be a trimer in aqueous solution, where the loss of secondary and tertiary structures is accompanied by loss of enzymatic activity in an irreversible unfolding process. The enzyme is more catalytically active on short acyl chain p-nitrophenyl esters (C2-C4) than on long acyl chain substrates (C8-C16). In the presence of modest amounts of organic solvents it retains hydrolytic activity and also exhibits an increase in thermal stability. Taken together, our results demonstrate features of the PmEst enzyme that indicate a potential application in industrial processes, particularly, when the use of polar organic solvents is required, such as the biodiesel synthesis or when tolerance to polar organic solvents is required, especially when substrate solubility is limited. The possible use of this enzyme for biotechnological applications is based on the preservation of its hydrolytic activity in the presence of polar solvents.

## Supporting Information

S1 File 3-D structural model of PmEst. (PDF)

S2 File PmEst fluorescence. (PDF)

S3 File Effect of pH in the PmEst structure. (PDF)

S4 File Thermal stability of PmEst in organic solvents. (PDF)

S1 Table A) Secondary structure content of PmEst estimated by SRCD and bioinformatics analyses. B) Lifetime analysis for PmEst in PBS as a function of temperature, using discrete model.
(PDF)
