# Supplementary material for: Environmental Factors Modulating the Stability and Enzymatic Activity of the Petrotoga mobilis Esterase (PmEst)
Source: PLoS One. 2016 Jun 28;11(6):e0158146. doi: 10.1371/journal.pone.0158146 (PMC4924860; doi:10.1371/journal.pone.0158146)
Supplement: S1 File — (PDF) [file pone.0158146.s001.pdf]

S1 Fig. 3-D structural model of PmEst.

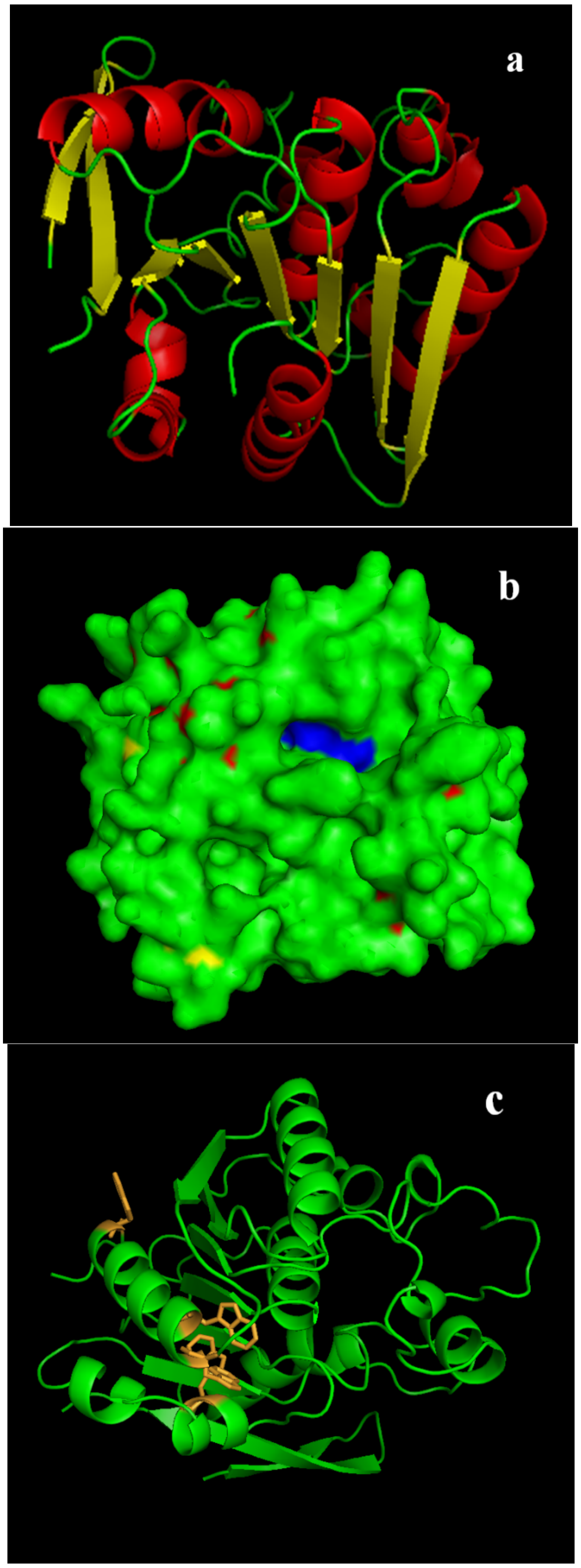

a) Model based on the homology modeling with the crystalline structure of tributyrin esterase (PDB ID: 2UZ0) in cartoon drawing; b) Residues of the esterase domain in PmEst are labeled in blue (on surface view); c) Position of the Trp residues in the 3-D structure of PmEst in cartoon drawing.
