# Supplementary material for: Environmental Factors Modulating the Stability and Enzymatic Activity of the Petrotoga mobilis Esterase (PmEst)
Source: PLoS One. 2016 Jun 28;11(6):e0158146. doi: 10.1371/journal.pone.0158146 (PMC4924860; doi:10.1371/journal.pone.0158146)
Supplement: S2 File — (PDF) [file pone.0158146.s002.pdf]

S2 Fig. PmEst fluorescence.

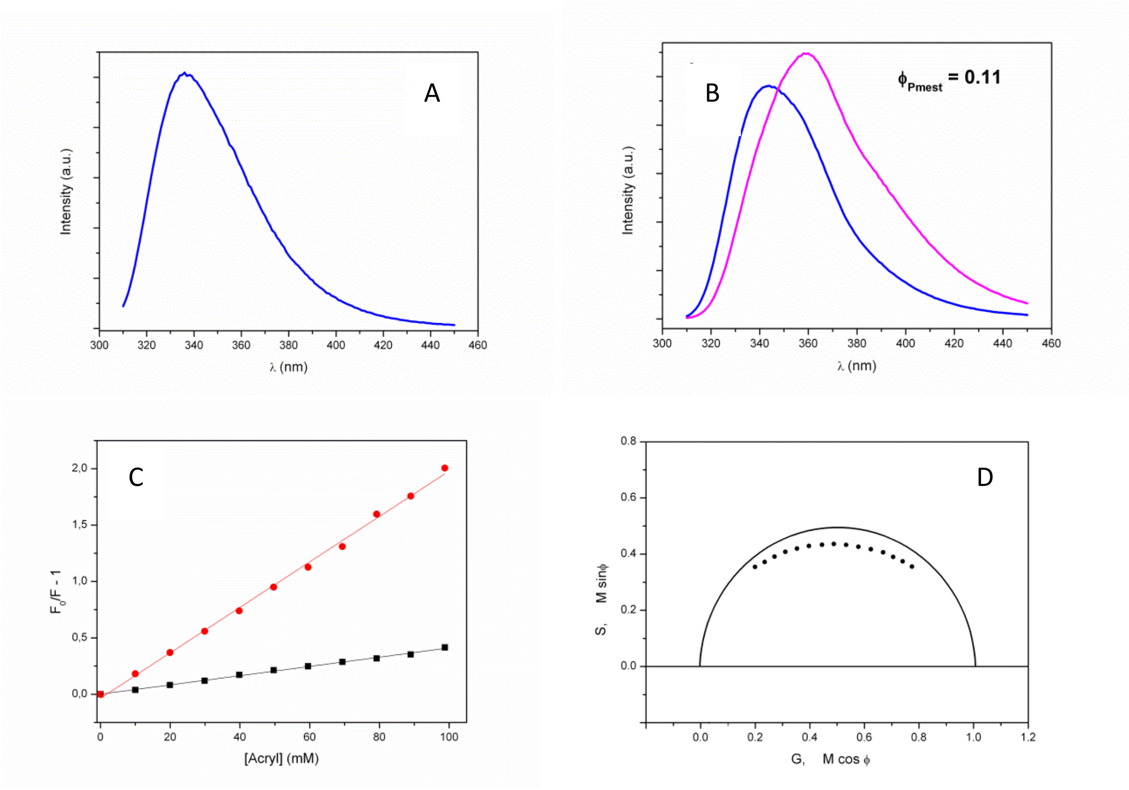

A) Emission spectrum and B) relative quantum yield determination of PmEst (blue), when NATA (magenta) was used as standard. C) Acrylamide quenching of the Trp residues in PmEst (black) and NATA (red) at 25º C, and D) Phasor plots of intensity decay of PmEst in aqueous solution (pH 7.0) at 25º C.
