# Supplementary material for: Environmental Factors Modulating the Stability and Enzymatic Activity of the Petrotoga mobilis Esterase (PmEst)
Source: PLoS One. 2016 Jun 28;11(6):e0158146. doi: 10.1371/journal.pone.0158146 (PMC4924860; doi:10.1371/journal.pone.0158146)
Supplement: S3 File — (PDF) [file pone.0158146.s003.pdf]

S3 Fig. Effect of pH in the PmEst structure.

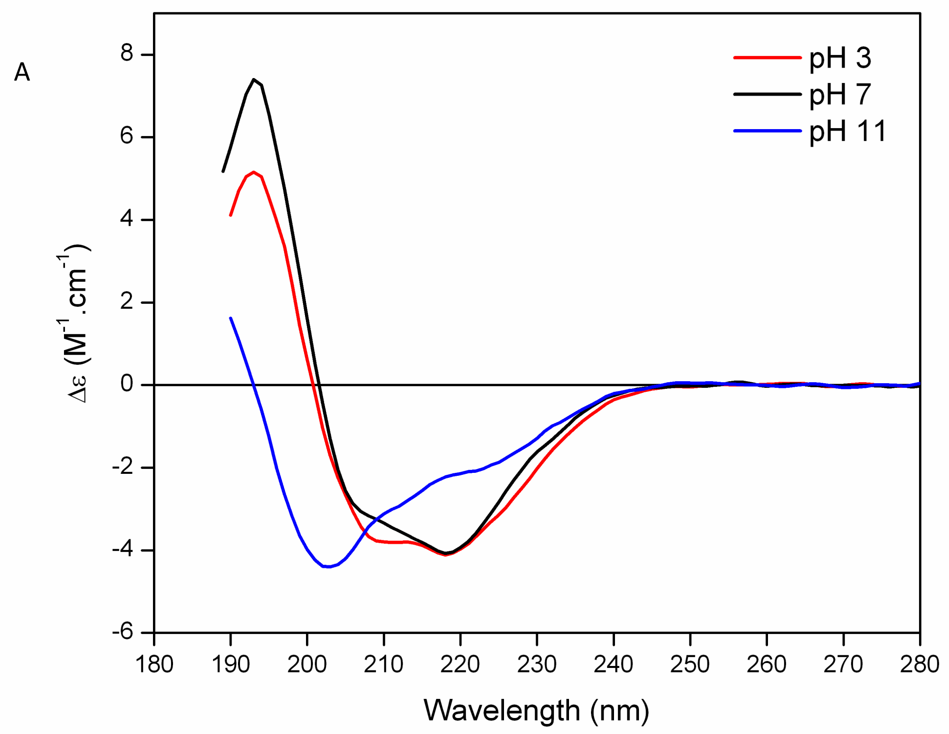

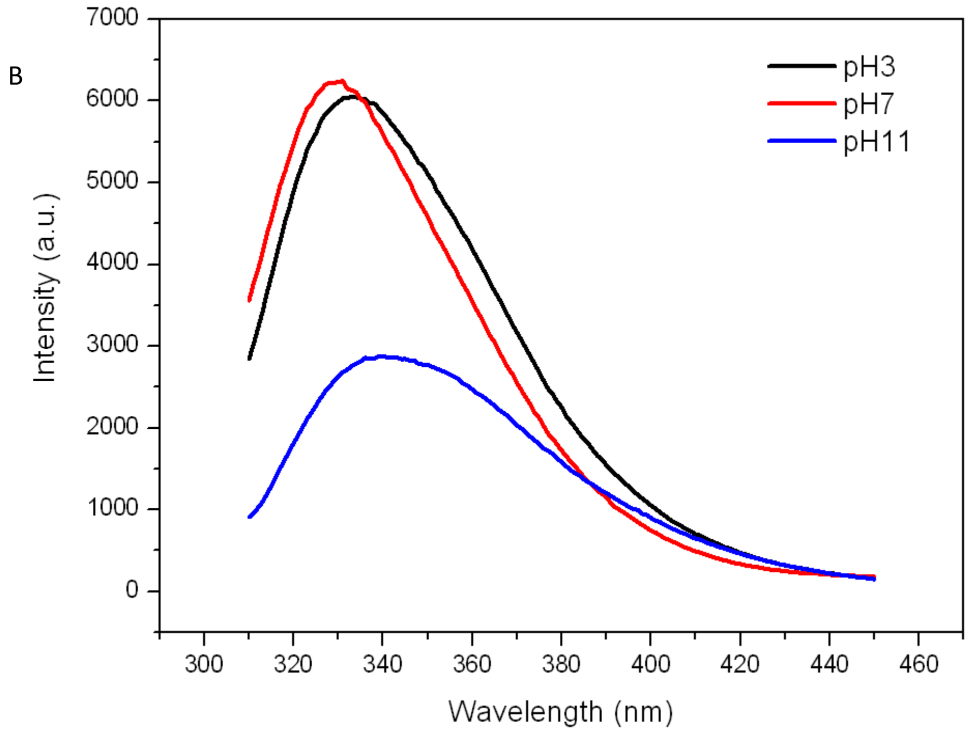

A) Circular dichroism and B) fluorescence spectra of PmEst in aqueous of different pH (3.0, 7.0 and 11).
