# Supplementary material for: Environmental Factors Modulating the Stability and Enzymatic Activity of the Petrotoga mobilis Esterase (PmEst)
Source: PLoS One. 2016 Jun 28;11(6):e0158146. doi: 10.1371/journal.pone.0158146 (PMC4924860; doi:10.1371/journal.pone.0158146)
Supplement: S4 File — (PDF) [file pone.0158146.s004.pdf]

S4 Fig. Thermal stability of PmEst in organic solvents.

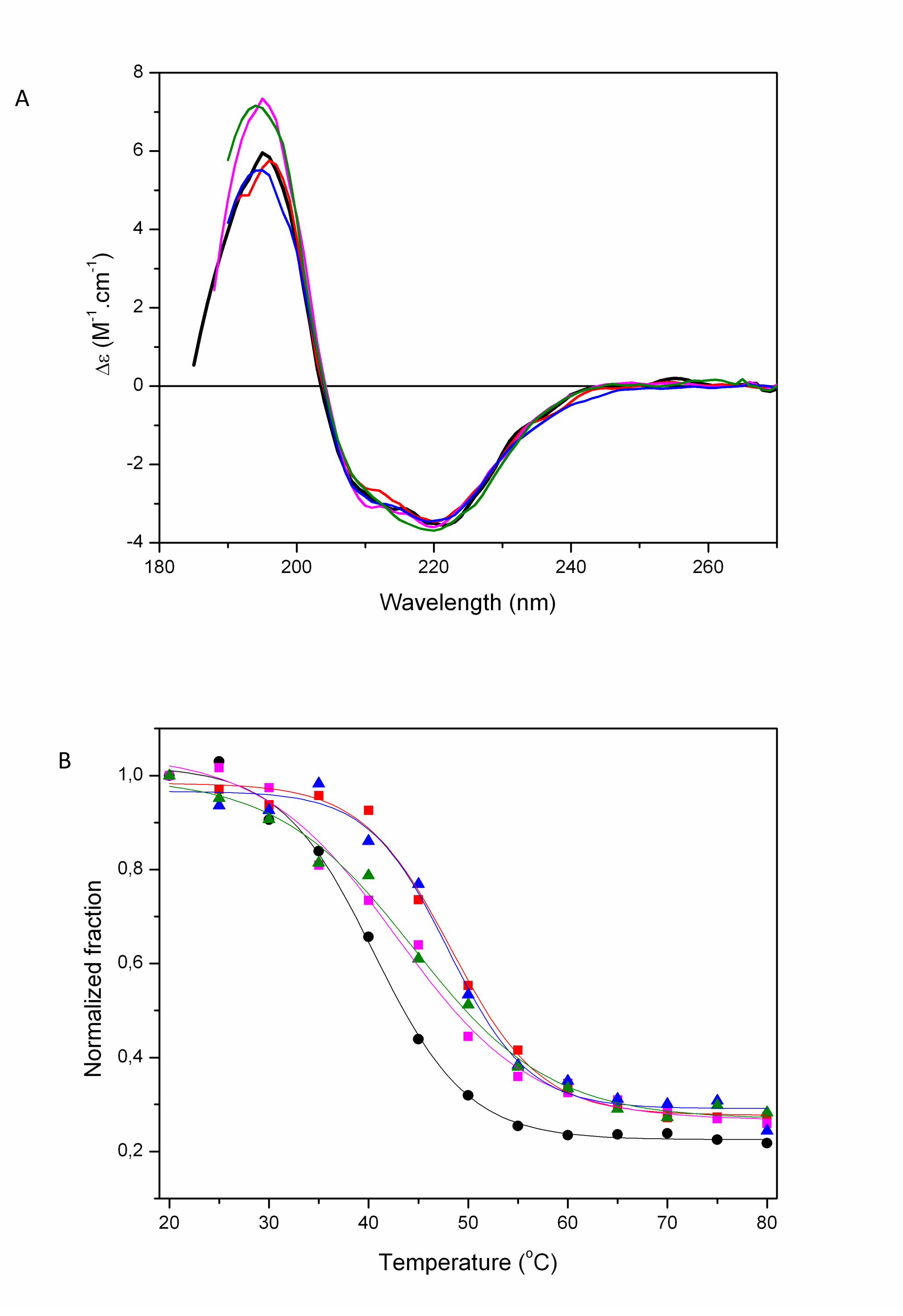

A) CD spectra of PmEst in 10 mM sodium phosphate buffer, pH 7.4 (black) and in the presence of 10% (red) and 20% (magenta) ethanol or 10% (blue) and 20% (green) propanol, at 20° C and B) Principal component analysis derived from the CD spectra from 20 to 80° C for the melting temperature of PmEst.
