# Supplementary material for: Environmental Factors Modulating the Stability and Enzymatic Activity of the Petrotoga mobilis Esterase (PmEst)
Source: PLoS One. 2016 Jun 28;11(6):e0158146. doi: 10.1371/journal.pone.0158146 (PMC4924860; doi:10.1371/journal.pone.0158146)
Supplement: S1 Table — A) Secondary structure content of PmEst estimated by SRCD and bioinformatics analyses. B) Lifetime analysis for PmEst in PBS as a function of temperature, using discrete model. (PDF) [file pone.0158146.s005.pdf]

S1 Table - Supporting Information

Table A. Secondary structure content of PmEst estimated by SRCD and bioinformatics analyses

	α-helix (%)	β-strand (%)	other (%)	NRMSD
Experimental SRCD *(25°C)*	31	26	43	0.064
PmEst *(3-D model)*	33	18	50	-
PmEst *(Psipred)*	33	17	50	-

NRMSD, normalised root mean square deviation

Table B. Lifetime analysis for PmEst in PBS as a function of temperature, using discrete model

PmEst	τ_1 (ns)	τ_2 (ns)	f_1	f_2	χ^2
15° C	5.58	1.90	0.37	0.63	0.80
25° C	5.28	1.67	0.35	0.65	0.91
35° C	4.95	1.41	0.35	0.65	0.96
45° C	4.73	1.23	0.36	0.64	0.98
